# The interaction of Lin28A/Rho associated coiled-coil containing protein kinase2 accelerates the malignancy of ovarian cancer

**DOI:** 10.1038/s41388-018-0512-9

**Published:** 2018-09-28

**Authors:** Yancheng Zhong, Sheng Yang, Wei Wang, Pingpin Wei, Shiwei He, Haotian Ma, Juan Yang, Qian Wang, Lanqin Cao, Wei Xiong, Ming Zhou, Guiyuan Li, Cijun Shuai, Shuping Peng

**Affiliations:** 1 Hunan Provincial Tumor Hospital and the Affiliated Tumor Hospital of Xiangya School of Medicine, School of Basic Medical Science, Central South University, Changsha, Hunan China; 2 Human Reproduction Center, Shenzhen Hospital of Hongkong University, Haiyuan 1 Road, Futian, Shenzhen China; 3 The Pathology Department of the Jining Medical University, Shan Dong, China; 4 The department of Gynecology of Xiangya Hospital, Central South University, Changsha, Hunan China; 5 Jiangxi University of Science and Technology, Ganzhou, 341000 China

**Keywords:** Tumour biomarkers, Metastasis, Cell migration

## Abstract

Ovarian cancer (OC) is the leading cause of death among women with gynecologic malignant diseases, however, the molecular mechanism of ovarian cancer is not well defined. Previous studies have found that RNA binding protein Lin28A is a key factor of maintain the pluripotency of stem cells, and it is positively correlated with the degree of several cancers (breast, prostate, liver cancer, etc). Our previous study shows that Lin28A is highly expressed in OC tissues and is involved in the regulation of OC cell biological behavior. In this study, we confirmed that high expression of Lin28A promoted the survival, invasion, metastasis, and inhibited the apoptosis of OC cells. Lin28A interacts with Rho associated coiled-coil containing protein kinase2 (ROCK2) but not ROCK1 and upregulates the expression of ROCK2 in OC cells. The binding sites of each other were identified by truncated mutations and Immuno-precipitaion (IP) assay. After knock down of ROCK2 in cells with high expression of Lin28A, the survival, invasion, metastasis was significantly inhibited and early apoptosis was increased in OC cells and OC xenograft in nude mice. Our experimental data also showed that knock down of ROCK2 but not ROCK1 inhibited the invasion by decreasing the expression of N-cadherin, Slug, β-catenin and increasing ZO-1 expression. Simultaneously, knock down of ROCK2 induced cell apoptosis by increasing cleaved Caspase-9,cleaved Caspase-7, and cleaved Caspase-3. Taken together, Lin28A regulated the biological behaviors in OC cells through ROCK2 and the interaction of Lin28A/ROCK2 may be a new target for diagnosis and gene therapy of OC.

## Introduction

Ovarian cancer (OC) is one of the gynecologic malignancies with the highest mortality rate [1]. OC accounts for 3% of the total incidence of gynecological cancer, the second only to endometrial cancer and cervical cancer, therefore the highest mortality of OC has been a serious threat to women’s lives all over the world [2, 3]. Since the ovaries are located deep in the pelvis, which is a hidden position, about 60% to 70% of patients were firstly diagnosed in the advanced stage of OC due to lack of early obvious or very particular symptoms [4, 5]. At present, the main treatment to OC patients was surgical treatment, radiotherapy, chemotherapy and molecular targeted therapy [6, 7]. Although there are some progress and improvement in short-term relief of patients with OC, the recurrence and metastasis rate are still very high. Moreover, the 5-year survival rate of OC patients who have surgical treatment and adjuvant chemotherapy has remained 20~ 40% [1, 8]. Due to lack of molecular mechanism on chemotherapy-resistance and early metastasis of OC, conventional treatments can hardly further improve the clinical effects. Increased efforts to get further understanding of the specific molecular mechanisms in OC are required for the development of new diagnostic and therapeutic strategies.

Lin28 has two paralogs, Lin28A and Lin28B, both of which containing two CCHC-zinc finger RNA-binding domain and one cold shock domain (CSD) [9]. Lin28 can modulate the levels of let-7 by CSD binding to the NGNGAYNNN (N = any base and Y = pyrimidine) sequence and CCHC-zinc finger can bind to the GGAG sequence which on the terminal loop of let-7 [10]. The sequence between the CHC-zinc finger and the CSD make it enable to bind all let-7 miRNA family members [10]. High expression of Lin28A are related to advanced human malignancies [11]. Increased studies are focused on expounding the effect of Lin28A/ let-7 in cancer [12]. However, our previous work showed that Lin28A can also recruit RHA to polysome and subsequently promote the bound Oct4 mRNA translation. It is highly expressed and co-expressed with Oct4 which showed co-relationship with poor prognosis of the patients with ovarian cancer [13, 14]. Lin28A modulates the function not only of miRNA but also of mRNA s as an RNA binding protein [9].

Rho associated coiled-coil containing protein kinase (ROCK), which appertains to the serine/threonine protein kinase family and is considered to be one of the most important downstream targets of Rho that is widely investigated [15, 16]. ROCK protein consists of N-terminal kinase domain, the coiled-coil region comprising a Rho binding domain (RBD) in the middle, and the pleckstrin homology (PH) domain as well ascysteine-rich domain (CRD) at the C-terninus [17]. ROCK family includes two subtypes, ROCK1 (ROKβ, p160-ROCK) and ROCK2 (ROKα), sharing 90% identity in amino acid sequence for full length and the coiled-coil region domain sharing least identity (55%) [18]. In the un-activated state, ROCK2 is self-suppressed by the PH domain, whereas when RhoA and RhoC are activated to bind to the RBD domain, the self-inhibition is disrupted and ROCK2 is activated [19, 20]. A lot of research have reported that ROCK2 plays an important role in invasion and metastasis in multiple cancers [21–23] and its high expression is correlated with poor prognosis in a variety of human cancers, including HCC, breast cancer and lung cancer [24, 25].

In this study, we demonstrated that high expression of Lin28A promoted cell survival, invasion and metastasis, and inhibited cell apoptosis of OC cells. And then we identified ROCK2 protein, which interacts with Lin28A through Immuo-precipitation (IP) assay and mass spectrometry. We found that the interaction with ROCK2 is necessary for Lin28A function. It was found that the binding domain of Lin28A and ROCK2 for each other, destroy of the interaction abated the function of promoting the survival and invasion of Lin28A. It is identified that Lin28A upregulated ROCK2 expression through protein synthesis or translation level. ROCK2 regulates the survival and cell invasion via β-catenin pathway through promoting the translocation of β-catenin into nucleus and inhibit the ubiquitination-dependent degradation. In summary, our finding have demonstrated a novel Lin28A/ROCK2 regulatory mechanism govern OC progression, which may provide a novel biomarker and potential therapeutic target for OC.

## Results

### Lin28A promotes OC cell proliferation by inhibiting cell apoptosis

It has been reported that Lin28A/Lin28B are expressed in normal embryogenesis and normal gonadal cells [26–28], but almost no expression in normal ovarian cells [29]. Up to now, the specific role and regulatory mechanism of Lin28A in OC remains unclear. In our previous studies, it was found that Lin28A high expression altogether with co-expression of Oct4 is associated with poor prognosis of ovarian cancer, while the roles and its mechanism on the development of OC remains unknown. Two ovarian cancer cell lines, A2780 with relatively low level of Lin28A and PA-1 with high level of endogenous Lin28A expression, respectively, were chosen for our study. We then established stable A2780 cells with full-length Lin28A cDNA inserted vector or empty vector as well as with shLin28A or shCtrl vector transfected PA-1 cells, respectively, named A2780 Ctrl, A2780 Lin28A, PA-1 shCtrl and shLin28A. Lin28A was expressed with high levels in A2780 cells with Flag-Lin28A vector and Lin28A was knockdown efficiently in PA-1 transfected with shLin28A vector.

**Fig. 1:**
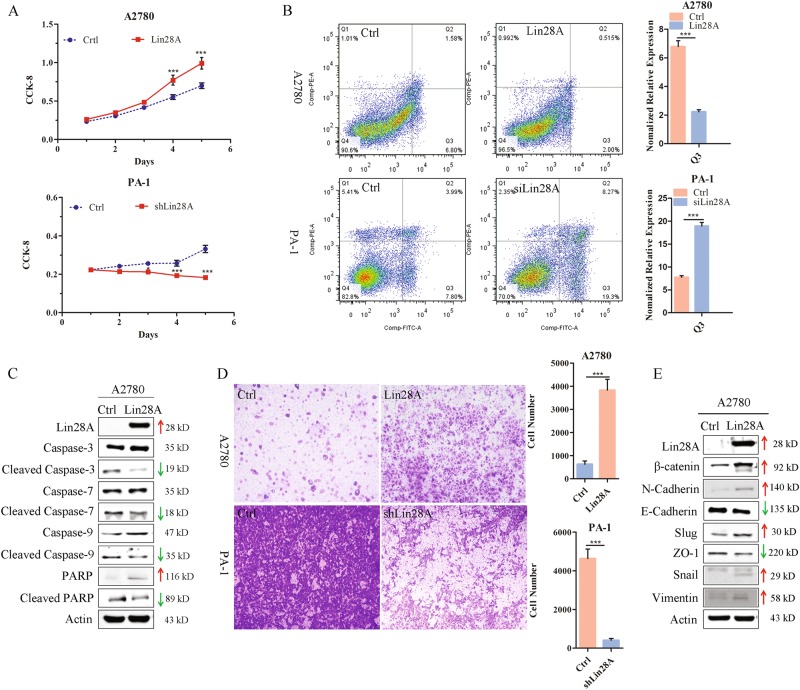
Lin28A accelerates the malignancy of ovarian cancer by promoting invasion and inhibiting cell apoptosis. **a** The effects of Lin28A on cell survival through CCK-8 analysis. *P < 0.05, ***P < 0.001. **b** Representative flow cytometry plots of Lin28A on cell apoptosis with Annexin V-EGFP and PI co-staining and statistical analysis. **c** Western blotting analysis of the levels of Lin28A and caspases (total and cleaved PARP, Caspase-9, −3 and −7). Actin acts as an internal reference. **d** The role of Lin28A in the invasion and metastasis of A2780 and PA-1 cells detected by transwell assay. Statistical analysis of invading cells. ***P < 0.001. Scale bars, 500 μm. **e** The effect of Lin28A on the levels of EMT-related molecules (Vimentin, Snail, Slug, N-cadherin, and β-catenin) and inhibitory molecule (ZO-1) examined by western blotting analysis

To investigate the effect of Lin28A on the survival of OC cells, CCK-8 assay and growth curve assay were performed. The results showed that the survival rate of A2780 Lin28A was higher than that of A2780 Ctrl, while the viability of PA-1 shLin28A was decreased significantly compared with PA-1 shCtrl (Fig. 1a), which suggested that Lin28A promoted the survival of OC cells. Next, we further explored whether Lin28A promote the survival of OC cells by affecting cell cycle or cell apoptosis. The results of cell cycle and apoptosis by flow cytometry showed that Lin28A did not affect the cell cycle, whereas the high expression of Lin28A could significantly inhibit cell early apoptosis compared with control (Fig. 1b). To further clarify the mechanism of regulation of Lin28A on cell apoptosis, we detected several apoptosis-related molecules by western blotting. The results showed that Lin28A can effectively inhibit the activated levels of cleaved Caspase-3, cleaved Caspase-7 and cleaved Caspase-9 protein, thereby inhibiting the DNA damage repair enzyme PARP from being cleaved in Lin28A overexpression group (Fig. 1c). Above results confirms that highly expressed Lin28A can promote cell survival through inhibiting cell apoptosis of OC cells.

### Lin28A promotes the invasion of OC cells

To test the role of Lin28A in cell invasion, we performed transwell invasion assay on the A2780 Lin28A or Ctrl and PA-1 shLin28A or shCtrl group. As the results shown, compared with control, the invading cells of A2780 Lin28A were significantly increased, whereas the invading cells of PA-1 shLin28A cells were obviously reduced (Fig. 1d), which indicates that Lin28A promote the invasion of OC cells. Epithelial–mesenchymal transformation (EMT) is a common phenotype of cancer cells, which display enhanced invasion ability. To further elucidate the mechanism of regulation of Lin28A on OC cell invasion, we detected several EMT-related molecules by western blotting analysis. Compared to A2780 Ctrl cells, the expression of Slug, vimentin, β-catenin, Snail and N-cadherin which promoted cellular invasion were upregulated in A2780 Lin28A; the inhibitory ZO-1 is downregulated (Fig. 1e).

### Lin28A interacts with ROCK2 but not ROCK1

**Fig. 2:**
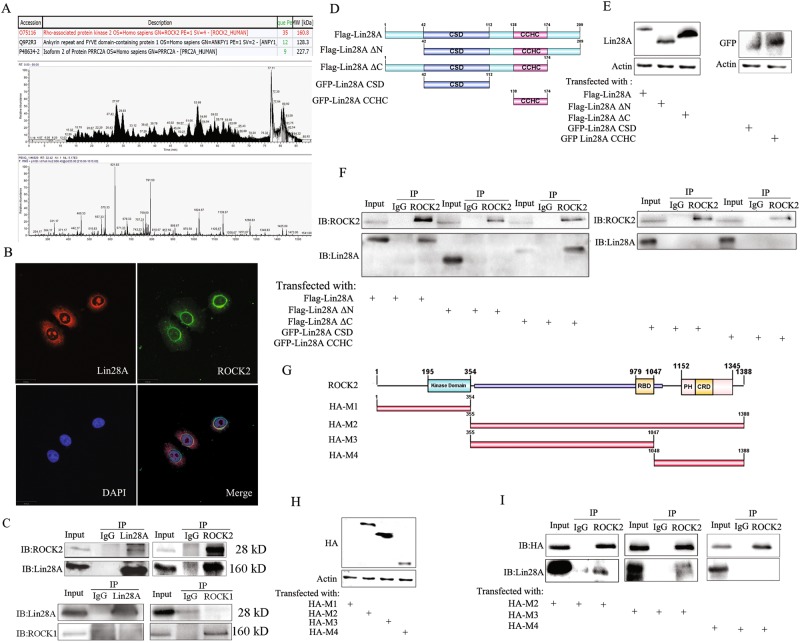
Lin28A interacts with ROCK2. **a** ROCK2 was included in the Lin28A-complex through IP combined with mass spectrometry. **b** Lin28A (Red) and ROCK2 (Green) were co-localized in the cytoplasm peri-nuclear. **c** IP experiment confirmed the interaction of Lin28A and ROCK2 (but not ROCK1) with anti-Lin28A and anti-ROCK2 (or anti-ROCK1) antibodies. **d** Domain-based Lin28A mutants were constructed successfully. **e** The expression of Lin28A mutants was expressed in HEK293 cells examined by western blotting analysis. **f** Lin28A mutants were transfected into HEK293 cells, respectively, IP with anti-ROCK2 antibody, western blotting with anti-Flag antibody. **g** Domain-based ROCK2 mutants were constructed successfully. **h** The expression of ROCK2 mutants was examined by western blotting analysis. **i** ROCK2 mutants were co-transfected with Flag-Lin28A vector in HEK293 cells, respectively, IP with anti-ROCK2 antibody, Western Blotting with anti-Flag antibody and anti-HA antibody

Since the above results confirmed that Lin28A played a role in inhibiting the apoptosis and promoting the invasion of OC cells, we further identified which proteins Lin28A interacts. Lin28A interacting protein complex was pulled down anti-Lin28A antibody through IP assay, then separated by PAGE and stained with AgNO3 solution. The unique bands for anti-Lin28A antibody compared to IgG were digested with enzymes and subjected to the mass-spectrometry analysis. The representative tentative peptide peaks were shown in Fig. 2a. Among the pull-down proteins, ROCK2 had the highest score, and the interaction between Lin28A and ROCK2 was further confirmed by IP assay and reverse IP as well as confocal microscopy analysis (Fig. 2b, c). Lin28A (Red) and ROCK2 (Green) were found to be located in the cytoplasm adjacent to the nucleus. As ROCK2 has high homology with ROCK1, we also investigated whether Lin28A interacts with ROCK1. IP assay confirmed the interaction of Lin28A and ROCK2 but not ROCK1 (Fig. 2c bottom). To map the binding sites of Lin28A and ROCK2, a series of domain-based truncated mutant constructs including Flag-Lin28A ΔN, Flag-Lin28A ΔC, GFP-Lin28A CSD and GFP-Lin28A CCHC (Fig. 2d). Then the mutants were transfected into HEK293 cells, and western blotting analysis showed that all of them can be expressed at high level (Fig. 2e). Then we transfected these mutants into HEK293, respectively, and IP assay showed that the full length of Lin28A and mutant Flag-Lin28A ΔC interacted with ROCK2, while the remaining mutants including Flag-Lin28A ΔN, GFP-Lin28A CSD and GFP-Lin28A CCHC did not interact with ROCK2 (Fig. 2f). These results indicated that Lin28A interact with ROCK2 by its N-terminus.

Similarly, four mutant constructs containing different domains of ROCK2 with HA tag were also established successfully, named as HA-M1, HA-M2, HA-M3, HA-M4 (Fig. 2g). These mutants were transfected into HEK293 cells and western blotting analysis showed that except HA-M1, the remaining mutants were expressed at high expression level (Fig. 2h). We co-transfected each mutant and Lin28A into HEK293, the proteins were extracted for IP experiment. The results of IP experiment showed that HA-M2 and HA-M3 interact with Lin28A, while HA-M4 did not interact with Lin28A (Fig. 2i). So we inferred that the binding sites of Lin28A on ROCK2 are located M3 region located 355~ 1047 amino acid of ROCK2. This amino acid segment of ROCK2 has lower homology with ROCK1, which is consistent with the previous hypothesis that Lin28A interacts with ROCK2 but not ROCK1.

### The interaction of Lin28A/ROCK2 promotes OC cell invasion and inhibits OC cell apoptosis

**Fig. 3:**
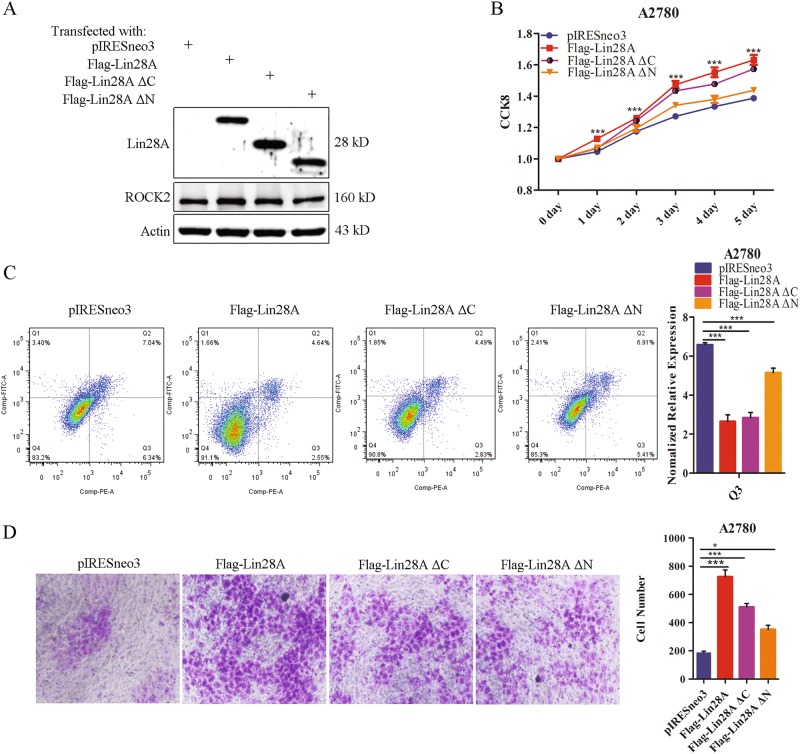
The interaction of Lin28A/ROCK2 promotes invasion and inhibits cell apoptosis. **a** The expression of Lin28A, Lin28A mutants and their role in the expression of ROCK2 was examined by western blotting analysis. **b** The effects of Lin28A and Lin28A mutants on cell survival through CCK-8 analysis. *P < 0.05, ***P < 0.001. **c** Representative flow cytometry plots of Lin28A and Lin28A mutants on cell apoptosis using Annexin V-EGFP and PI co-staining. ***P < 0.001. **d** The role of Lin28A and Lin28A mutants in the invasion and metastasis of A2780 and PA-1 cells detected by transwell assay. Statistical analysis of invading cells. ***P < 0.001, *P < 0.05. Scale bars, 500 μm

Since we have confirmed that the N-terminus of Lin28A interact with ROCK2, then we explored the roles of the interaction between Lin28A and ROCK2. Then the mutants Flag-Lin28A ΔN, Flag-Lin28A ΔC and Flag-Lin28A were transfected into A2780 cells respectively, and Western Blotting analysis showed that all of them can be expressed at high level. We found that the expression of ROCK2 in the cells with Lin28A full-length and Lin28A ΔC was higher than that of Lin28A ΔN, indicating that the interaction of Lin28A/ROCK2 is required for Lin28A function in the expression of ROCK2 (Fig. 3a).

To examine the effect of the Lin28A/ROCK2 interaction on the survival of OC cells, CCK-8 assay and growth curve assay were performed. The results showed that the survival rate of Flag-Lin28A and Flag-Lin28A ΔC was higher than that of empty vector and Flag-Lin28A ΔN, the viability of Flag-Lin28A ΔC is not good as to Flag-Lin28A, although it has the binding site with ROCK2, suggesting that the deletion region may play some roles somehow (Fig. 3b). At least this suggested that the interaction really can promote the viability of OC cells. The results of cell apoptosis by flow cytometry showed the high expression of Flag-Lin28A and Flag-Lin28A ΔC could significantly inhibit cell apoptosis compared to vector pIRESneo3 and Flag-Lin28A ΔN (Fig. 3c). This further confirms that the interaction of Lin28A/ROCK2 can promote cell survival through inhibiting cell apoptosis of OC cells.

To detect the roles of the Lin28A/ROCK2 interaction in cell invasion, we performed transwell invasion assay for pIRESneo3, Flag-Lin28A ΔN, Flag-Lin28A ΔC and Flag-Lin28A group, respectively. Compared with pIRESneo3, the migrated-through cells of the Flag-Lin28A, Flag-Lin28A ΔC and Flag-Lin28A ΔN group were significantly increased. As expected, the migrated-through cells in Flag-Lin28A ΔN are much less than that in Flag-Lin28A ΔC and full-length Lin28A group (Fig. 3d). These experimental data demonstrated that the interaction between Lin28A and ROCK2 can promote the invasion ability of OC cells.

### Lin28A upregulates the protein level of ROCK2 in OC cells

**Fig. 4:**
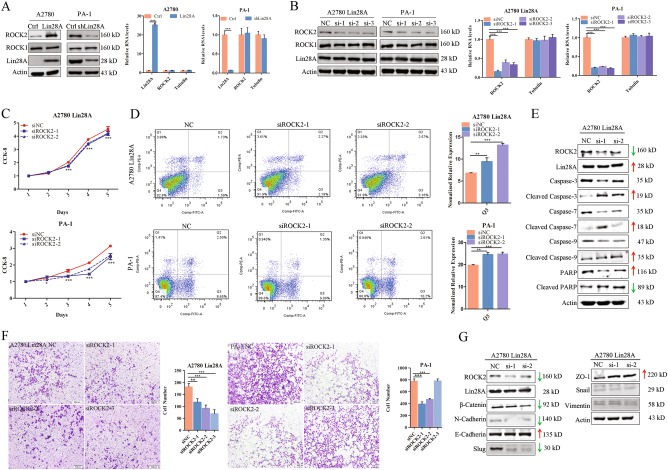
Lin28A promotes the malignancy of OC by upregulating ROCK2. **a** Lin28A upregulated the expression of ROCK2 in A2780 and PA-1 cells at protein level by western blotting but not mRNA level by qRT-PCR analysis. **b** ROCK2 expression was knocked down by siRNA interference at protein level (left) and mRNA level (right) in A2780 Lin28A and PA-1 cell, respectively. **c** CCK-8 analysis showed siROCK but not siNC decreased the survival rate of A2780 Lin28A (top) and PA-1 cells (bottom), respectively. *P < 0.05, ***P < 0.001. **d** Knock down of ROCK2 by siRNA increased the apoptosis of A2780 Lin28A and PA-1 cell through flow cytometry. **P < 0.01, ***P < 0.001. **e** Knock down ROCK2 by siRNA increased the cleavage of Caspase-9, −3 and −7, decreased the cleavage of PARP, Actin acts as an internal reference. **f** Knockdown of ROCK2 deceased the invasive ability of A2780 Lin28A and PA-1 cells measured by transwell assay. Statistical analysis of invaded cells, **P < 0.01, ***P < 0.001, respectively. Scale bars, 500 μm. **g** Knockdown of ROCK2 deceased the expression of invasion-related molecules and upregulated adhesion-associated molecules such as E-cadherin and ZO-1

We measured the expression of Lin28A and ROCK2 by western blotting and qRT-PCR analysis. The results revealed that Lin28A upregulated the protein level of ROCK2, while Lin28A did not affect the mRNA level of ROCK2 in OC cells (A2780 and PA-1 cells) (Fig. 4a).

After that we designed and synthesized three ROCK2 siRNAs, they were transiently transfected into PA-1 and A2780 Lin28A cells. ROCK2 was knockdown efficiently at the protein level and mRNA level in PA-1 and A2780 Lin28A cells by western blotting and qRT-PCR analysis (Fig. 4b). However, knockdown of ROCK2 does not affect the expression level of Lin28A (Fig. 4b) as well as ROCK1 which suggested that siRNAs against ROCK2 were specific to ROCK2 but not ROCK1. It is hypothesized that Lin28A is the upstream regulatory molecule of its interacting protein, ROCK2, and upregulates the protein level of ROCK2 in OC cells.

### Knock down of ROCK2 suppresses the survival through promoting cell apoptosis of OC cells

We detected that Lin28A upregulated the expression of the protein levels of ROCK2 in OC cells. In order to further determine whether Lin28A promotes the cell migration and inhibits cell apoptosis through the interaction with ROCK2, we studied the effect of ROCK2 knock down in OC cells. The results of CCK-8 assay showed that when ROCK2 was knocked down, the cell survival was decreased compared to siNC in both A2780 Lin28A and PA-1 cells (Fig. 4c). Similarly, we also evaluated the differences of cell cycle and apoptosis with flow cytometry after ROCK2 knocked down. The results showed that ROCK2 knockdown did not affect the cell cycle, whereas ROCK2 knock down could significantly promote cell apoptosis compared with siNC group (Fig. 4d). To further support this result, we examined apoptosis molecules using western blotting analysis with or without ROCK2 knockdown. The results showed that the activated level of cleaved Caspase-3, cleaved Caspase-7, cleaved Caspase-9 and cleaved PARP protein were upregulated in the ROCK2 knockdown group (Fig. 4e). It further confirms that knockdown of ROCK2 decreases OC cells survival through inducing cell apoptosis.

### Knock down of ROCK2 suppresses the invasion of OC cells

To evaluate the effect of ROCK2 in cell invasion, we performed transwell invasion assay for A2780 Lin28A and PA-1 cells with or without ROCK2 knock down. Compared with siNC, the invading cells of A2780 Lin28A/siROCK2 group and PA-1/siROCK2 group were significantly reduced (Fig. 4f), which indicated that knock down of ROCK2 can inhibit the invasion ability of OC cells. In order to further elucidate the mechanism, several EMT-related molecules were examined with western blotting analysis, and the results showed that, siROCK2 caused the downregulation of β-catenin, Slug and N-cadherin, which promoted cellular invasion, and also promoted the expression of ZO-1, which inhibited cell invasion (Fig. 4g). The above results confirmed that Lin28A played a role in promoting cell survival and invasion of OC cells by upregulating the expression of interaction protein ROCK2.

### Lin28A/ROCK2 promotes the EMT by activating the pathway of Wnt/β-catenin

**Fig. 5:**
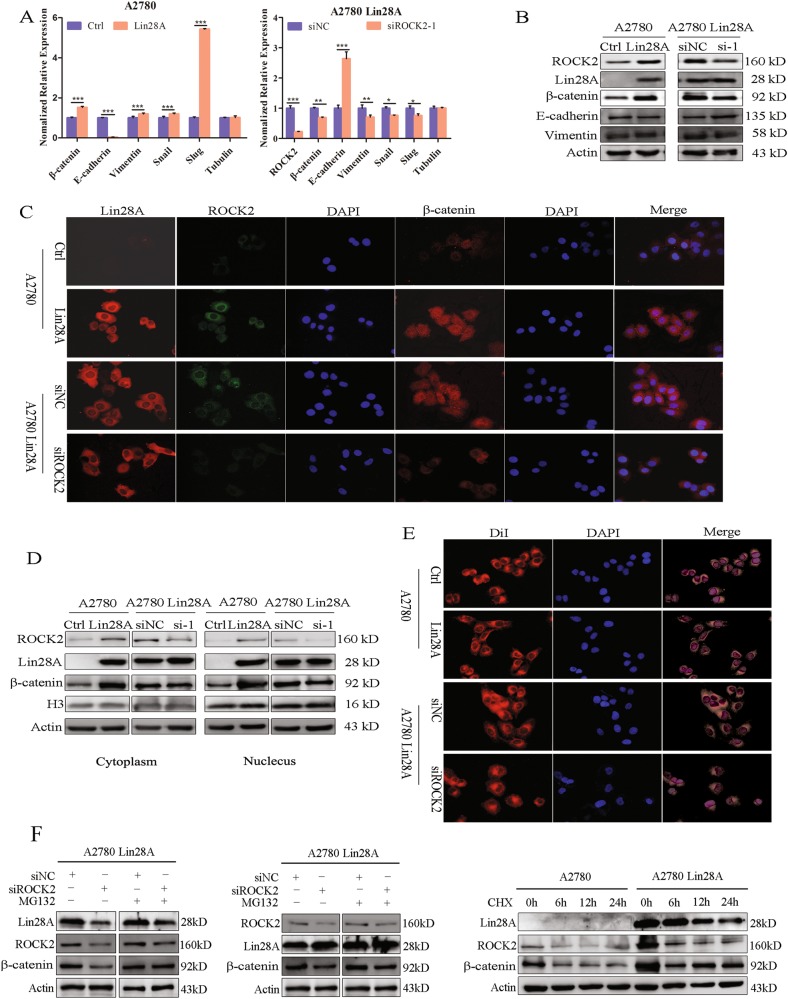
Lin28A/ROCK2 promotes the EMT of OC cells by activating Wnt/β-catenin pathway. **a**, **b** Western blotting and qRT-PCR analysis the EMT-related molecules in A2780 Ctrl, A2780 Lin28A and A2780 Lin28A siNC, A2780 Lin28A siROCK2 cells. **P < 0.01, ***P < 0.001. **c** Lin28A (red)/ROCK2 (green) can promote β-catenin into the nucleus by immunofluorescence experiments. **d** Lin28A/ROCK2 can promote β-catenin into the nucleus by detecting the expression of β-catenin in the nucleus and cytoplasm. H3 acted as a nucleus protein reference. Actin acted as an internal reference. **e** Lin28A/ROCK2 can promote OC cells to undergo EMT by DiI (red) staining experiments. **f** OC cells treated with MG132 (100 ng/ml) and CHX (100 μg/ml), respectively, and western blotting analysis the expression of Lin28A, ROCK2 and β-catenin. Actin acted as an internal reference

We found that high expression of Lin28A increased the expression of β-catenin, and knockdown of ROCK2 also reduced the expression and nucleus translocation of β-catenin. β-catenin is an important member of Wnt/β-catenin signaling pathway, and a large number of studies have shown that activated the signaling pathway of Wnt/β-catenin can promote EMT in cells and further promote cell invasion and metastasis. We supposed that Lin28A and ROCK2 can play a role in promoting cell invasion and metastasis by promoting the activation of Wnt/β-catenin signaling pathway. Firstly, we confirmed Lin28A/ROCK2 can promote the expression of β-catenin by qRT-PCR and Western Blotting analysis (Fig. 5a, b). The translocation of β-catenin into the nucleus from cytoplasm is the most important step of Wnt/β-catenin signaling pathway activation. Lin28A/ROCK2 can increase the protein level of β-catenin and promote it into the nucleus through con-focal microscopy (Fig. 5c). The nucleus and cytoplasm protein extraction also confirmed the results (Fig. 5d). Finally, we can see Lin28A/ROCK2 can promote cell morphology from epithelial to mesenchymal transition through the fluorescence staining of DiI membrane protein (Red) (Fig. 5e), which indicated that Lin28A/ROCK2 promotes the EMT of OC cells by activating the pathway of Wnt/β-catenin.

To further determine the regulatory relationship among Lin28A, ROCK2 and β-catenin, we treated OC cells with MG132 (a potent reversible cell-permeable proteasome inhibitor) and CHX (Cycloheximide, a denovo protein synthesis inhibitor), respectively. MG132 treatment will inhibit the proteasome-dependent degradation of ROCK2. However, siLin28A still decreased the ROCK2 protein level upon MG132 treatment, indicating Lin28A doesn’t affect the protein degradation of ROCK2 and Lin28A probably regulated ROCK2 expression at protein translation level. However, MG132 and CHX treatment all changed the roles of Lin28A or ROCK2 on β-catenin protein level, indicating that Lin28A and ROCK2 can simultaneously promote the protein synthesis of and inhibit the protein degradation of β-catenin (Fig. 5f).

### Lin28A and ROCK2 are highly expressed in OC tissue and negatively associated with OC prognosis

**Fig. 6:**
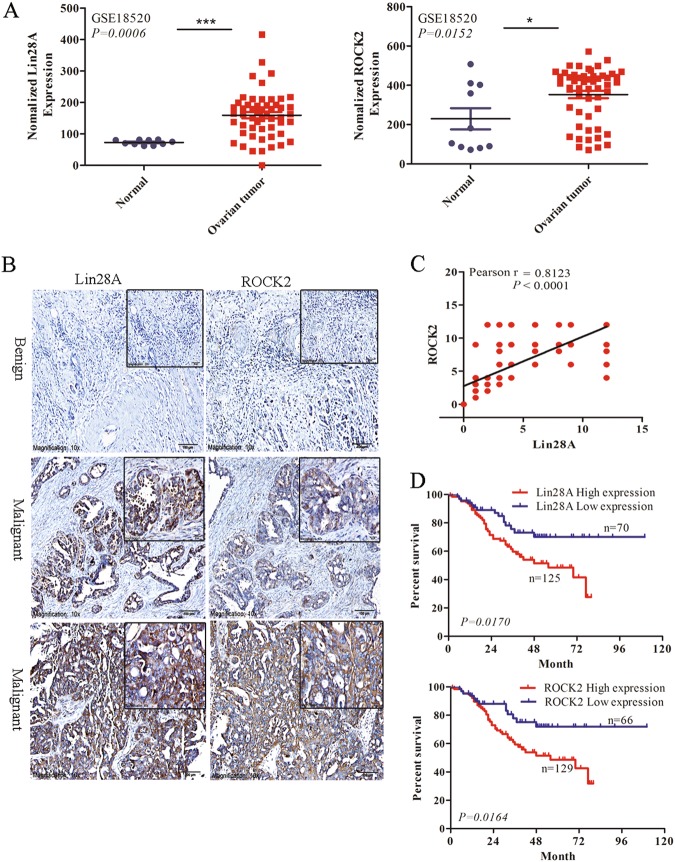
The expression of Lin28A and ROCK2 were positively correlated and associated with poor prognosis in OC. **a** Both the mRNA expression of Lin28A (left) and ROCK2 (right) in OC were upregulated compared that of paracancerous adjacent normal ovarian epithelium tissues in the database GES18520. *P* = 0.0006 and *P* = 0.0152, respectively. **b** Representative images for the expression of Lin28A and ROCK2 was shown in OC tissue by immunohistochemistry analysis. Scale bar, 100 μm, 20 μm, respectively. **c** Pearson correlation analysis showed that the expression of Lin28A was positively correlated to that of ROCK2 in OC tissues. Pearson *R* = 0.8123, *P* < 0.0001. **d** Ten year survival analysis suggested the expression of Lin28A (top) and ROCK2 (down) negatively were co-related to the prognosis of OC patients using Kaplan–Meier survival analysis. *P* = 0.0170 and *P* = 0.0164, respectively

We analyzed the expression of Lin28A and ROCK2 in 55 cases of OC tissues and 10 cases of paracancerous normal epithelial tissues in the GEO database (GSE18520). We found that both Lin28A and ROCK2 were highly expressed in OC tissues compared with paracancerous normal epithelium tissues in the GEO database (GSE18520) (Fig. 6a). In order to further verify that the expression correlation of Lin28A and ROCK2 in OC tissues, we measured the Lin28A and ROCK2 expression through immunohistochemical analysis of OC tissue microarray containing 195 cases of OC patients. Representative photographs showed that the expression of ROCK2 varies with Lin28A expression (Fig. 6b). The correlation analysis of Lin28A and ROCK2 demonstrated that the expression of Lin28A and ROCK2 was significantly positively correlated (Pearson r = 0.8123, *P* < 0.0001) (Fig. 6c). Finally, it is found that the OC patients with the high expression of Lin28A and ROCK2 have lower 10-year survival rate compared with those patients with low expression of Lin28A and ROCK2. The results showed that the high expression of ROCK2 and Lin28A were associated with poor prognosis in patients with OC (Fig. 6d).

### Lin28A promotes the tumor growth and metastasis of OC xenograft in vivo

**Fig. 7:**
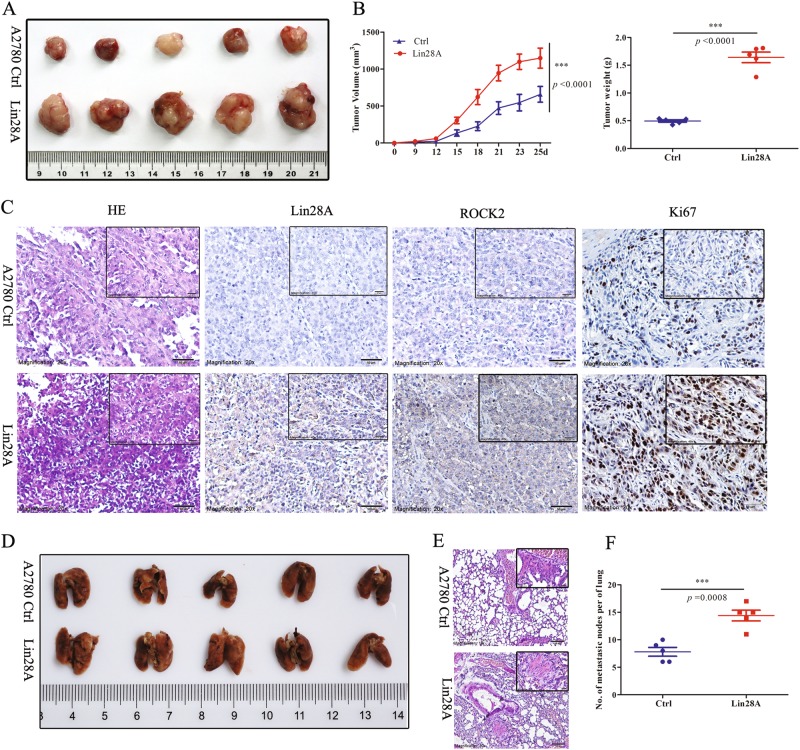
Lin28A promoted the tumor growth and metastasis of OC xenograft in vivo. **a** The photograph of stripped tumor from A2780 Ctrl (top) and A2780 Lin28A (down) group (*n* = 5) 38 days after transplantation. **b** The curve of the tumor volume (left) and final weight (right) from Ctrl and Lin28A group. *P* < 0.0001. **c** Paraffin sections were stained with H&E solution and immunohistochemistry analysis for Lin28A, ROCK2 and Ki67 antibodies. Scale bars, 50 μm, 20 μm. **d** A photograph of lungs from the mice transplanted with A2780 Ctrl (top) and A2780 Lin28A (down) group. **e** H&E staining of the lungs of mice transplanted with A2780 Ctrl and A2780 Lin28A. Scale bars, 50 μm, 20 μm. **f** The lung metastases of Lin28A group were more than that of Ctrl group. *P* = 0.0008

Since we have confirmed the role of Lin28A in OC cells and OC tissues, we also tried to investigate whether it can affect the development of OC in vivo experiments. A2780 Ctrl/Lin28A cells were transplanted in the either of axilla of Balb/c nude mice. The tumor volume and size of subcutaneous xenografts were measured every week. The mice were killed on 31d after A2780 Ctrl/Lin28A cells were initially injected. It was found that the tumors growing in the Lin28A group were bigger in size than those growing in the Ctrl group statistically significantly, which indicated that Lin28A promoted the growth of OC xenograft in vivo (Fig. 7a). It was observed that the tumor growth was remarkably increased by Lin28A, the final volume of tumors in Lin28A group was about (1.746 ± 0.26) times bigger than that in Ctrl group and the weight of tumors in Lin28A group was about (3.32 ± 0.079) times larger than that in Ctrl group (Fig. 7b). IHC staining indicated a lower expression level of Lin28A and ROCK2 in the tumor tissues of A2780 Ctrl group while a higher expression level of Lin28A and ROCK2 in A2780 Lin28A group. Lin28A and ROCK2 were mainly located in cytoplasm (Fig. 7c). The expression level of Lin28A and ROCK2 was positively associated with the cell proliferative nuclear antigen Ki67 which represented of the rate of cell proliferation (Fig. 7c).

For the invasiveness and metastasis of A2780 Ctrl/A2780 Lin28A cells, white metastasis nodules were observed in lung surface of both Ctrl and Lin28A nude mice group (Fig. 7d). Similarly, the metastatic nodes in A2780 Lin28A group (13.8 ± 2.0 nodules) are much more than that of A2780 Ctrl group (8.0 ± 1.7 nodules) (Fig. 7e, f). The H&E staining for paraffin sections of lungs in Lin28A group significantly increased number of lung metastasis nodes than that of Ctrl group (Fig. 7e). These results confirmed that Lin28A increased not only the tumor growth but also the metastasis of ovarian cancer xenograft in vivo.

### Knock down of ROCK2 inhibits the growth and metastasis of OC xenograft in vivo

**Fig. 8:**
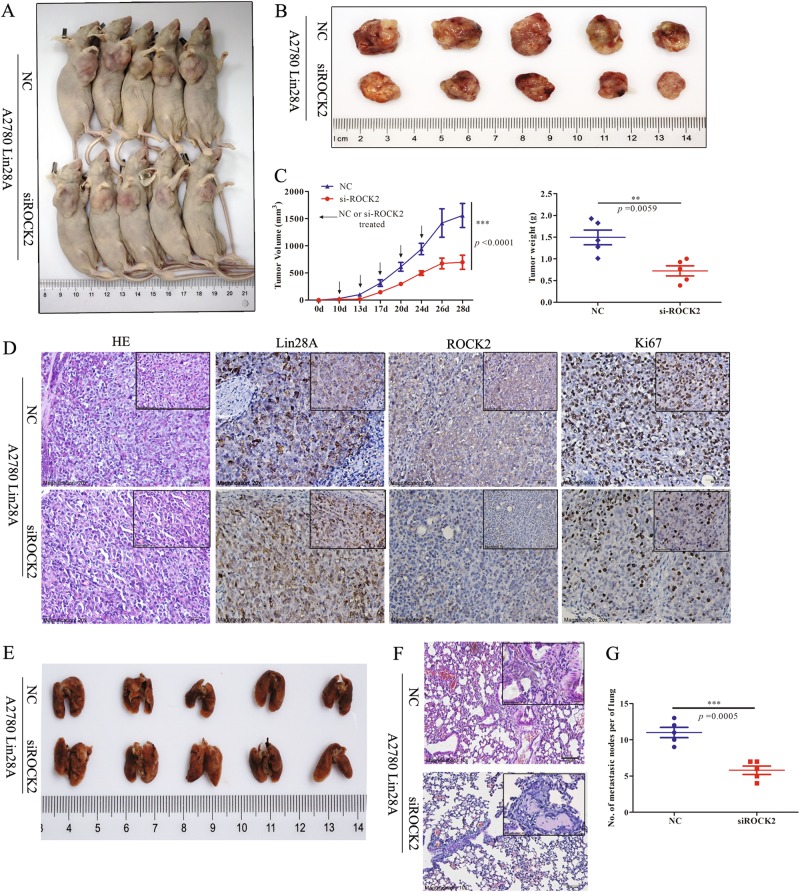
Knock down of ROCK2 by siROCK2 restrains the tumor growth and metastasis of OC xenograft in vivo. **a**, **b** A photograph of nude mouse and tumors from NC (top) and siROCK2 (bottom) group. **c** Schema for the analysis of tumor volume (left) and weight (right) from NC and siROCK2 group. *P* < 0.0001 and *P* = 0.0059. **d** H&E staining and immunohistochemistry analysis for Lin28A, ROCK2 and Ki67 expression in each group. Scale bar, 100 μm, 20 μm, respectively. **e** A photograph of lungs from NC (top) and siROCK2 (bottom) group. **f** H&E staining of the lungs of NC (top) and siROCK2 (bottom) group. Scale bar, 100 μm, 20 μm, respectively. **g** The lung metastases of siROCK2 group was less than that of NC group. *P* = 0.0005

In order to verify the role of ROCK2 in vivo, we designed and synthesized 2′-O -Methyl-conjugated-5′Cholesterol (2′Ome-5′Chol) -modified siROCK2 and negative control (NC) for in vivo experiments. The equal A2780 Lin28A cells (5.0 × 10^7^) were transplanted subcutaneously into either axilla of Balb/c nude mice and OC xenografts formed. On day 10 after tumor transplantation, 2 nmol/mouse siROCK2 or NC was intratumorally injected into tumors (6 times in total). Mice were killed 38d after A2780 Lin28A cells were initially injected. It was found that the tumor sizes of the siROCK2 group was smaller than those of NC, which indicated that lower expression of ROCK2 caused smaller OC xenografts in vivo (Fig. 8a, b). The tumor growth was remarkably decreased by siROCK2. The tumor growth was remarkably decreased by siROCK2, the final tumor volume of siROCK2-treated mice was (44.72 ± 0.32)% of NC-treated mice, and the tumor weight was (48.39 ± 0.15)% of the NC-treated mice (Fig. 8c). IHC staining indicated a lower expression level of ROCK2 injected with siROCK2 while a higher expression level with ROCK2 injected with NC (Fig. 8d). The expression level of ROCK2 was positively related to the cellular nuclear antigen Ki67 which represented of the rate of tumor growth (Fig. 8d).

White metastatic nodules were observed on lung surface of nude mice both in NC and siROCK2 group (Fig. 8e); however, the number of the white metastatic nodules of the lung in siROCK2 group (7.5 ± 2.1 nodules) significantly is less than that of NC group (12.3 ± 1.9 nodules) (Fig. 8g). The H&E staining for lung tissues showed that siROCK2 decrease the metastasis of OC xenografts to the lungs in nude mice (Fig. 8f). Similarly, these results indicated that ROCK2 knockdown decreased not only the tumor growth but also the metastasis and invasion of OC cells in vivo.

## Discussion

In this study, we identified ROCK2 as interacting protein of Lin28A, an important pluripotency factor in OC cells. Both Lin28A and ROCK2 were highly expressed in OC tissues. Kaplan–Meier analysis revealed that high expression of Lin28A and ROCK2 was both correlated with poor clinical outcomes in OC patients and positively correlated with each other. Lin28A interacts with ROCK2 and upregulates the expression of ROCK2, and promoted the survival of OC cells by downregulating the expression of apoptosis-related molecules and inhibiting their apoptosis in OC cells. At same time we also found that Lin28A promoted the invasive ability of OC cells through regulating the expression of EMT-related molecules.

**Fig. 9:**
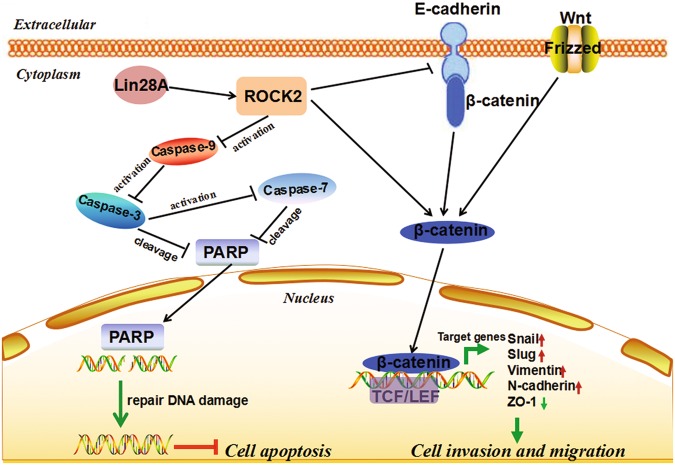
A working model for Lin28A/ROCK2 inhibits cell apoptosis and promotes invasion of OC cells. Lin28A upregulates ROCK2 to inhibit apoptosis by inhibiting the activation of Caspase-9, −3 and −7, and then inhibit the degradation of the DNA repair enzyme PARP, so that PARP could repair the damaged DNA, and ultimately inhibits the apoptosis in OC cells. Lin28A upregulates ROCK2, and then increases the expression of β-catenin, Snail, Slug, Vimentin and N-cadherin, which promotes cellular invasion, and inhibits the expression of ZO-1, which inhibits cellular invasion, and finally promotes the invasion and migration in OC cells

The two-way negative regulation of Lin28A/let-7 pathway has been gained much attention. Lin28A blocks the maturation of let-7 precursor, leading to its downregulation [30]. It was reported that in neural progenitor cells, Lin28A has been shown to upregulate IGF-2 to inhibit the caspase-dependent apoptosis [31]. Suggesting that highly expressed Lin28A in OC may inhibit the activation of caspase-3. In our study, we found that Lin28A can regulate the expression of the interact protein ROCK2 to promote the malignant development of OC, however, there is no binding sites for let-7 in the mRNA of ROCK2. This suggested that Lin28A promote the malignant development of OC does not dependent on the classic Lin28A/let-7 negative regulatory network, and we found a novel regulatory network by which Lin28A promoted the malignant development of OC. The highly expressed Lin28A could inhibit the activation of Caspase-9, Caspase-3 and Caspase-7, and then inhibit the degradation of the DNA repair enzyme PARP, so that PARP could repair the damaged DNA, and ultimately inhibited the apoptosis in OC cells. It was reported that ROCK2 regulated the development of colorectal cancer, prostate cancer, glioma, breast cancer, and bladder cancer [32–34]. There are also studies confirmed that drug sensitivity increased after knockdown of ROCK2 in OC cells, and interrupting ROCK pathway can promote apoptosis in hepatocellular carcinoma cells [34]. This indicated that ROCK2 can regulate cell apoptosis. Our study found that after ROCK2 knock down the apoptosis increased in OC cells, by promoting the activation of Caspase-9, Caspase-7 and Caspase-3, thereby increasing the degradation of PARP, and ultimately reduce the ability of repair DNA damage (Fig. 9).

High metastasis and recurrence are the leading causes of death in OC patients, various studies have shown that when cells undergo EMT, cells lose their polarity, then tight junctions become loose, these make cancer cells possess the ability of migration and invasion [35, 36]. The Wnt/β-catenin pathway is one of the most important pathways involved in EMT [37]. We found Lin28A can upregulate the expression of β-catenin, Snail, Slug, vimentin and N-cadherin which promoted cellular invasion; also can inhibit the expression of ZO-1, which inhibited cellular invasion. Several studies reported ROCK2 can promote cell migration and movement [38–40]. In addition, in tumor cells, Rho/ROCK2 signaling pathway activation leads to increase cytoskeleton dynamics and cell invasion [41]. Moreover, our study confirmed that knock down ROCK2 can cause downregulation of the expression of β-catenin, Slug and N-cadherin which promoted cellular invasion; also promoted ZO-1 expression, which inhibited cellular invasion. This also further confirmed that Lin28A targets ROCK2 to promote invasion by affecting the EMT-related molecules in OC cells (Fig. 9).

In this study, we found that Lin28A can accelerate the malignancy of OC by upregulating its interacting protein ROCK2. Our previous studies reported that Lin28A can promote the translation of its bound target mRNA levels by recruiting RHA helicase (RHA) to polysomes [42]. We also found that Lin28A can also bind to ROCK2 mRNA by RNA-protein precipitation assay, and inferred that Lin28A promote the protein synthesis of ROCK2 which is consistent to our finding that it does not affect the degradation of ROCK2 but affect the protein translation. This explains why Lin28A does not affect the mRNA level of ROCK2, but it can upregulate the protein expression of ROCK2. We infer that Lin28A upregulated the ROCK2 expression through recruiting RHA to the ROCK2 mRNA and promoted the translation, which shared similar mechanism on Oct4, which demonstrated in our previous publications [42].

The Wnt/β-catenin pathway plays an important role in the metastasis and invasion of various cancers of various cancers [43]. In this study, we found that Lin28A upregulates the protein level of ROCK2 and promote the expression of β-catenin effectively and its entry into the nucleus. ROCK2 was reported to stabilize the expression of β-catenin by inhibiting its ubiquitination degradation [44]. In OC, we also found that Lin28A can stabilize the expression of β-catenin by inhibiting its degradation in ROCK2 dependant manner, in addition, Lin28A can also promote its protein synthesis and increase the nucleus translocation of β-catenin.

Finally, we further determined that Lin28A promoted tumor growth and metastasis through the interaction with ROCK2 in vivo experiments. Although we are not yet able to identify the detailed mechanisms of Lin28A on regulating the apoptosis and metastasis of OC cells, our findings suggested that the ectopic expression of Lin28A/ROCK2 may be used as markers for the prognosis of OC patients and the interaction of Lin28A/ROCK2 may be targeted for the treatment of OC patients. In conclusion, our findings provide novel mechanistic insight into the role of Lin28A/ROCK2 in the OC development and progression. Targeting Lin28A/ROCK2 may be a potential effective therapeutic approach for OC patients.

## Materials and methods

### Cell lines and cell culture

Human OC cells A2780 were purchased from ATCC, PA-1 was purchased by the Chinese Academy of Medical Sciences, Institute of Basic Medical Sciences, Beijing Union Medical College Cellular Resource Center. The cell lines were identified by STR profiling in 6 months. To obtain a stable expression of Lin28A cell lines, we constructed pIRES-Flag-Lin28A expression vector, the reagent material comprises: pIRESneo3 vector plasmid (PT3645-5, 631621), Pyrobest® DNA Polymerase (TaKaRa, DR500A), Not I (TaKaRa, 1166 A), EcoR I (TaKaRa, 1040 A), Glue recovery kit, CloneEZ^®^ Recombinant cloning kit (Kingsui biotechnology, L00339). After the recombinant plasmid was sequenced, the plasmid vector was transfected into A2780 cells by Lipofectamine™ 2000 transfection (Invitrogen, 11668–027) and the cell clone was screened by G418 and expanded to obtain Lin28A overexpressing cell line, which was named A2780 Lin28A. And we obtained Lin28A silenced cell line by PA-1 lentivirus infection.

HEK293 and A2780 cells were cultured in DMEM medium (Gibco, USA) containing 10% fetal bovine serum (FBS) (Gibco, USA). PA-1 cells were cultured in RPMI 1640 medium (Gibco, USA) containing 10% FBS. All cells were cultured at 37 °C in 5% CO_2_ cell incubator. The cells were digested with trypsin EDTA solution A (BI, Israel) and cryopreserved with FBS containing 10% DMSO (Sigma-Aldrich, USA).

### Immunohistochemistry and immunostaining evaluation

Select 195 cases of patients aged 17–80 years (mean age 57 years) with OC from January 2008 to December 2014 in the Affiliated Hospital of Jining Medical University (Shandong, China). All clinical samples were collected with OC patients’ informed consents.

Sample sections were incubated with following primary antibodies: anti-Lin28A (Abcam) (1:500), anti-ROCK2 (Abcam) (1:600), anti-Ki67 (BBI Life Sciences) (1:300). All immunohistochemical sections were independently scored by three different investigators.

The sum of the staining intensity score and the positive rate score was taken as the IHC score. Staining intensity score: No coloring test 0 points; light yellow count 1 point; brown yellow count 2 points; dark yellow count 3 points. Positive rate score: No positive cells: 0 points; positive cells number greater than 25%: 1 point; positive cells number between 26 and 50%: 2 points; positive cells number between 51% and 75%: 3 points; positive cells more than 75%: 4 points. Multiply the two scores and divide into four levels according to the total score. The negative expression (−) is 0, the weak expression (+) is 1–4, the intermediate positive expression (++) was 5–8, and the strong positive expression (+++) was 9–12.

### Bioinformatics analysis

Selected an OC gene microarray data from GEO database (GEO accession: GSE18520). We analyzed the mRNA expression levels of Lin28A and ROCK2 between normal ovarian tissues and OC tissues from GSE18520 data.

### Quantitative RT-PCR (qRT-PCR)

Total RNA was isolated from cells using Trizol Reagant (Life Technologies, USA). Then cDNA was synthesized according to the protocol of Revert Aid First SYBR Green PCR Kit (Thermo Fisher Scientific, USA). The qRT-PCR was detected by the CFX96 real-time PCR detection system (Bio-rad, USA) following the protocol of 2 × SYBR Green qPCR Master Mix (Bimake, USA). Actin and Tubulin were used as an internal control.

### Immunoblotting and immunoprecipitation assay

A RIPA mixed buffer containing protease inhibitor and phosphatase inhibitor was used as the cell lysate. Total cell extracts were separated by SDS-PAGE and transferred to PVDF Membrane (Milipore, Germany) and incubated with primary antibodies: anti-Lin28A, anti-ROCK2 (Abcam, USA), EMT Antibody Sampler Kit (Cell Signaling Technology, USA), Apoptosis Antibody Sampler Kit (Cell Signaling Technology, USA), and Actin (ABclonal, USA) as the internal reference. Membrane was then incubated with HRP-conjugated anti-mouse or anti-rabbit IgG antibodies according to the source of primary antibody. Then membrane was imaged with the ChemiDoc MP System (Bio-Rad, USA).

For immunoprecipitation assay, total cell lysates were prepared with a buffer including 1% Triton X-100, 150 mM NaCl, 1% Na-deoxycholate, 10 mM Tris-HCl, 5 mM EDTA, 0.1% SDS, protease inhibitor and protease inhibitor. The protein lysates were incubated with anti-ROCK2 antibody or anti-Lin28A antibody. The protein A/G PLUS-Agarose beads were used to immunoprecipitate proteins linked to the primary antibody. Western Blotting analysis was then performed as described.

### Cell migration assay and CCK-8 assay

Stable OC cell lines or transfected OC cells were used for analysis. 8.0 × 10^4^ OC cells were resuspended with 200 μl DMEM medium without FBS and seeded in the upper chamber of Transwell Chamber (Costar, USA), which was matrigel-covered, simultaneously, 700 μl DMEM medium including 15% FBS was slowly injected to the lower layer. When the cells invade through the chamber and fall into the lower lower layer, we terminate the culture. The invading cells were then fixed with 4% paraformaldehyde and stained with crystal violet solution (Sangon Biotech Co, China) about 2 min, the cells on the upper chamber were wiped, and photographed under the Optical Microscope (Olympus, Japan). We took 5 fields of view for each group of cells and counted the invading cells by Image-Pro Plus software.

For CCK-8 assay, stable OC cell lines or transfected OC cells were seeded in a 96-well plate at 2000 cells per well. After 6 h culture at 37 °C in 5% CO_2_ cell culture incubator, each well was incubated with 10 μl CCK-8 (Sigma-Aldrich, USA) for 2 h in 37 °C cell culture incubator, then the absorbance was measured at a wavelength of 450 nm by Paradigm Dectection Platform (BECK MAN, CA) and normalized to that of 0 day; then culture was further carried out for 5 days, and the absorbance was measured at a fixed time point for 5 consecutive days. After the results were summarized, the growth curve of OC cells was plotted in GraphPad Prism 5.0.

### Tumor xenografts in mice

In an in vivo experiment, we randomly divided 20 female BALB/c nude mice which aged 4–5 weeks and weighing 16–18 g into 4 groups (n = 5 per group). All animal experiments were carried out within the guidelines with the Animal Care and Use Committee of Central South University. All groups of BALB/c nude mice were injected subcutaneously, one group was subcutaneously injected with 100 μl DMEM medium containing 5.0 × 10^7^ A2780 cells, while another three groups were injected subcutaneously with 100 μl DMEM medium containing 5.0 × 10^7^ A2780 Lin28A cells. When the length (*L*) and width (*W*) of tumors grew about 5 mm × 5 mm (about 10 day) of that the two groups which were injected with A2780 Lin28A cells, 2 nmol of NC or siROCK2 (RiboBio Co, China) was injected to the introtumorous twice a week, for a total of 3 weeks. At the same time, the length and width of the tumors were measured twice a week with a micrometer. And using the formula *V* = 1/2 × *L* × *W*^2^ calculated the volume (*V*: mm^3^) of tumors. Then all tumor tissues and lung tissues were fixed with 4% paraformaldehyde solution for ~36 h, then embedded by paraffin and sectioned.

### Apoptosis assay and cell cycle assay

Stable OC cell lines or transfected OC cells were used for analysis. Apoptosis assay was examined using the annexin VFITC/PI Apoptosis Detection Kit (BIOBOX, China) following to the reagent’s instructions. And for cell cycle detection, OC cells were re-suspended by 300 μl PBS, then 700 μl cooled ethanol was added to the cell suspension, and was fixed for at least 12 h at −20 °C. Follow the steps in the reagent’s instructions for the Cell Cycle Staining Kit (Multi Science, China).

### Statistical analysis

All data statistics were presented as mean ± standard deviation (Mean ± SD). The analysis between the two groups of data was performed using the *t* test; the analysis between the three groups of data was analyzed by one-way ANOVA; the survival of OC patients was analyzed by Kaplan–Meier. All statistical data were performed by the software Graph Pad Prism 5. The statistical results obtained from the independent and randomized three replicate experiments, and the *P* value was less than 0.05 (*P* < 0.05) were considered to be statistically significant.

## Electronic supplementary material

Supplementary figure legends

Figure S1

Figure S2

Figure S3

Figure S4

Table S1

Table S2
