# Supplementary material for: The interaction of Lin28A/Rho associated coiled-coil containing protein kinase2 accelerates the malignancy of ovarian cancer
Source: Oncogene. 2018 Sep 28;38(9):1381–97. doi: 10.1038/s41388-018-0512-9 (PMC6372474; doi:10.1038/s41388-018-0512-9)
Supplement: Supplementary file 1 — Supplementary figure legends [file 41388_2018_512_MOESM1_ESM.doc]

**The interaction of Lin28A/****Rho associated coiled-coil containing protein kinase2 accelerates the malignancy of ovarian cancer**

Yancheng Zhong^1#^,Sheng Yang^2#^, Wei Wang^3^, Pingpin Wei^1^, Shiwei He^1^, Haotian Ma^1^, Juan Yang^1^, Qian Wang^4^, Lanqin Cao^4^, Wei Xiong^1^ , Ming Zhou^1^,Guiyuan Li^1^, Cijun Shuai^5^, Shuping Peng^1*^

**Supplementary figure legends**

**Figure S1 The cell lines in which Lin28A was stably expressed were established successfully.** (A-B) Lin28A was over-expressed in A2780 cells and knocked down by shLin28A lenti virus system in PA-1 cells at the protein and mRNA level.

**Figure S2 The effects of Lin28A and ROCK2 on cell cycle.** (A) The role of Lin28A in cell cycle of A2780 cells by Flow cytometry analysis. (B) Knockdown of ROCK2 didn't affect the cell cycle of A2780 Lin28A cells through Flow cytometry analysis.

**Figure S3 The protein domains of ROCK2 and ROCK1.** (A) The domains of ROCK2 protein. (B) The domains of ROCK1 protein.

**Figure S4 Lin28A protein binds to ROCK2 mRNA in OC cells examined by RNA-protein Immunoprecipitation assay.**

**Table S1 Lin28A and ROCK2 expression in normal ovarian tissue and OC tissue（Data from GSE18520).**

**Table S2 The clinical information of OC tissues (N=195).**
