# Supplementary figures and images for: The interaction of Lin28A/Rho associated coiled-coil containing protein kinase2 accelerates the malignancy of ovarian cancer
Source: Oncogene. 2018 Sep 28;38(9):1381–97. doi: 10.1038/s41388-018-0512-9 (PMC6372474; doi:10.1038/s41388-018-0512-9)

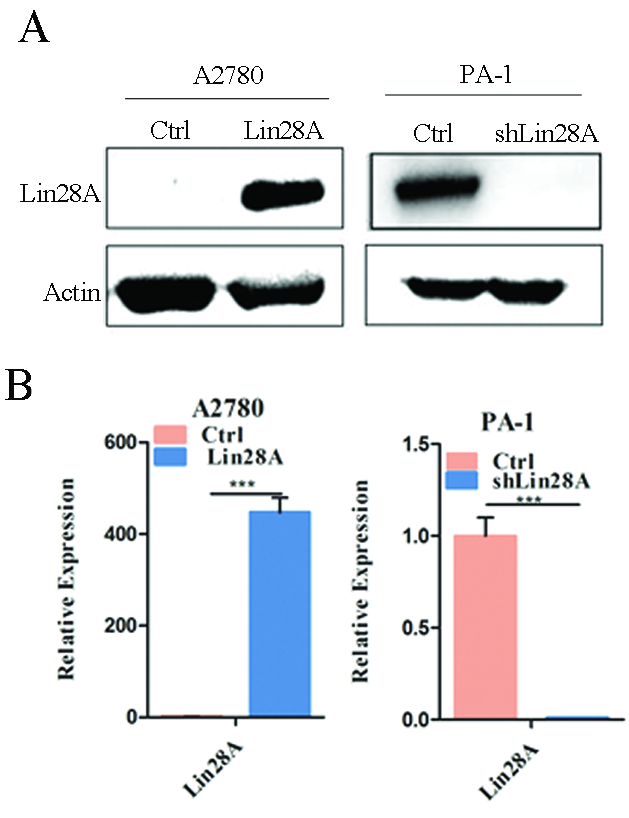

Supplement: Supplementary file 2 — Figure S1 [file 41388_2018_512_MOESM2_ESM.tif]

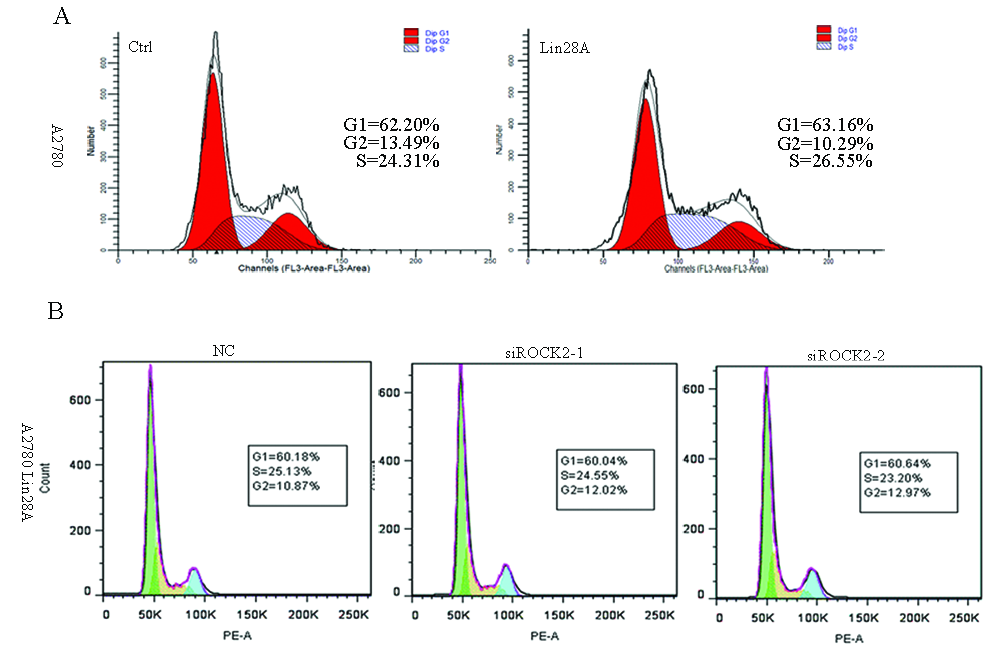

Supplement: Supplementary file 3 — Figure S2 [file 41388_2018_512_MOESM3_ESM.tif]

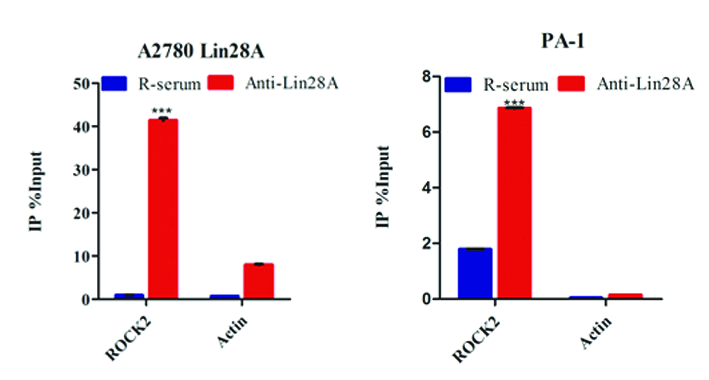

Supplement: Supplementary file 5 — Figure S4 [file 41388_2018_512_MOESM5_ESM.tif]
